# Temporal Trajectories in Sleep, Temperature Trends, Cardiorespiratory, and Activity Metrics Measured via Oura Ring During Pregnancy: Large-Scale Observational Analysis

**DOI:** 10.2196/80213

**Published:** 2025-10-27

**Authors:** Rebecca Adaimi, Nina Thigpen, Alicia Clausel, Neta Gotlieb, Ketan Patel, Massimiliano de Zambotti

**Affiliations:** 1 Ouraring Inc. San Francisco, CA United States

**Keywords:** pregnancy, consumer wearables, sleep, activity, heart rate, postpartum

## Abstract

**Background:**

Pregnancy and childbirth involve significant health challenges, including preventable maternal deaths, severe complications, and disparities tied to social determinants, emphasizing the need for improved maternal care. Pregnancy could benefit from a more comprehensive, continuous care model that captures dynamic changes and enhances maternal-fetal outcomes.

**Objective:**

This large-scale, real-world, high-density study aims to use wearable data to investigate maternal biobehavioral trajectories for pregnancies leading to loss, preterm, and term births, exploring how demographic factors like age and body mass index (BMI) affect these trajectories.

**Methods:**

Retrospective observational analysis of pregnancies from a sample of 10,318 and 18- to 51-year-old female Oura Ring users (324 preterm births, 5039 term births, 4955 pregnancies ending in loss before 20 weeks of gestation). Oura biobehavioral data were analyzed across a 64-week window encompassing 8 weeks prepregnancy, through pregnancy, and post partum, via generalized estimating equation (GEE) statistical modeling.

**Results:**

Gestational age emerged as a significant factor across all domains among term pregnancies (*P*<.001). During the first trimester, participants experienced marked sleep changes, peaking around week 9 and characterized by more time in bed (+30 min), asleep (+15 min), and awake (+15 min) compared with prepregnancy. Metrics declined and stabilized in the second trimester; by the third trimester, time in bed returned to baseline, while sleep remained reduced and wakefulness elevated. At birth, time in bed and wakefulness peaked, and sleep duration reached its minimum, with nighttime wake exceeding 3 SDs above baseline. Temperature changes were more pronounced, sustained, and occurred earlier than sleep changes—becoming evident by week 4, peaking at +0.3 °C above baseline by week 9, and showing a steady decline until birth. A secondary, modest increase (+0.1 °C) was observed near birth, followed by a decline postpartum. Heart rate (HR) increased steadily, peaking at +10 bpm above baseline at week 32, while HR variability declined by >15 milliseconds in a mirrored pattern. Respiratory rate peaked around week 9 and declined thereafter. Step count declined in the first trimester, with a ≈2000-step reduction at around week 8. After a slight rebound midpregnancy, activity declined again, reaching its lowest point near birth, with >2500 fewer steps than prepregnancy. Age and BMI showed significant but modest interaction effects (all *P*<.01). In pregnancies ending in loss, deviations emerged up to 2 weeks prior. Time in bed decreased starting ≈2 weeks before loss (*P*<.001), followed by reductions in sleep duration (*P*<.001), temperature trends (*P*<.001), respiratory rate (*P*=.019), HR (*P*=.005), and awake time (*P*=.033).

**Conclusions:**

These findings highlight the complex dynamics underlying changes in participants’ sleep, temperature trends, cardiorespiratory, and activity data throughout pregnancy, involving extensive adaptations. A deeper focus on normative changes can advance maternal-fetal medicine, improve clinical outcomes, and address scientific gaps.

## Introduction

More than 4 births occur every second of every day worldwide. In the United States alone, more than 10,000 babies are born (live births) each day [1]. According to the World Health Organization, approximately 287,000 women died during pregnancy or childbirth in 2020, equating to one maternal death every 2 minutes [2]. Data from the 2017-2019 Maternal Mortality Review Committees in the United States showed that over 80% of pregnancy-related deaths (occurring during pregnancy, delivery, and up to a year postpartum) were determined to be preventable [3]. Beyond mortality, millions of women experience severe maternal morbidity, including life-threatening complications such as hemorrhage, preeclampsia, and infections. For example, in the United States, as many as 60,000 women per year are affected by severe maternal morbidity, most of which could have been avoided with timely interventions [4]. Miscarriage (loss of a fetus before the 20th week of pregnancy) and stillbirth (loss of a fetus after 20 weeks of pregnancy) are common and represent significant emotional and physical trauma. Importantly, maternal health outcomes are deeply tied to social determinants of health. Women in underserved communities face higher risks due to limited access to health care, systemic inequalities, and a lack of prenatal care.

Pregnancy represents a highly complex physiological state, characterized by extensive biological, hormonal, and behavioral adaptations that optimize maternal and fetal development. Current prenatal care is largely centered on assessing health and screening for disease at discrete time points during scheduled visits, focusing on key physical markers such as blood pressure, weight, and fetal growth. In the United States, standard prenatal care varies based on a woman’s risk profile. Standard prenatal care globally includes relatively few touchpoints compared with the length of pregnancy and the significant physiological and emotional changes that occur. Postpartum, care and connection often drop off, with sometimes only a single visit after delivery. Those considered high-risk may require more frequent visits and ad hoc interventions depending on the severity and progression of pregnancy-associated factors, but the care, nevertheless, is brief and infrequent [5].

While valuable, this episodic approach often fails to capture the dynamic and continuous nature of physiological, behavioral, and psychosocial changes throughout pregnancy. Critical fluctuations or early signs of atypical trajectories may go unnoticed between visits. Notably, it overlooks individual trajectories relative to one’s baseline, potentially missing significant deviations that, while within population norms, may still indicate important risks or shifts in health status. This limitation underscores the need for a more comprehensive, individualized, longitudinal model of prenatal care, incorporating continuous monitoring and integrating emerging biobehavioral and contextual data. Such an approach would provide a deeper understanding of pregnancy as an evolving process and improve maternal-fetal outcomes.

Traditional maternal health research has been instrumental in shaping our understanding of pregnancy, providing critical insights into fetal development, maternal physiology, and the factors contributing to pregnancy outcomes. Through clinical assessments and structured studies, this body of work has established foundational knowledge and improved care practices.

Thanks to the advancements in sensor technology and digital health, multisensor consumer wearables (ie, smartwatches, smart rings, and other health trackers) in particular, present an opportunity to complement and extend traditional research approaches. However, this line of work is still in its infancy, and more research is needed to understand their role in supporting the reproductive life course [6-8]. By enabling passive, large-scale monitoring of biobehavioral data—such as sleep, temperature trends, cardiorespiratory, and activity patterns—wearables could provide a dynamic and real-time perspective on maternal health throughout pregnancy. Wearables demonstrate feasibility in estimating ovulation compared with calendar-based fertility tracking [9], presenting a potential avenue to support women’s fertility journeys. Grant and Smarr [10] showcase the possibility of passive early pregnancy detection through continuous analysis of distal skin temperature, while others suggest the use of a wearable to proxy the clinically estimated delivery date [11,12]. Other studies showcase the potential to use wearables in relation to pregnancy risks (eg, classification of healthy pregnant vs nonpregnant women) [13] and complications, including gestational diabetes [14], gestational hypertension [15], preterm labor [11,16], and postpartum depression [17].

The maternal health market is evolving rapidly, with a growing focus on digital health solutions and consumer-centric innovations to address the diverse needs of pregnant women and new mothers. While the role of consumer wearable in maternal health is still unclear, initial feasibility for continuous high-resolution passive monitoring of pregnancy using a multisensor consumer wearable device has been shown by Keeler et al [18]. In that study, biobehavioral data collected via the Oura Ring Gen2 and questionnaire data were analyzed from 120 pregnancies, including 97 full-term pregnancies and 23 cases of pregnancy loss. Two additional recent studies evaluating signals before, during, and after pregnancy highlight changes across 3 distinct physiological states. A retrospective analysis of sleep data from 2540 Fitbit users in the US and Canada examined patterns 12 weeks before pregnancy, throughout gestation, and during the first 20 weeks postpartum [19]. Similarly, an evaluation of 757 pregnancies for Apple Watch users described trends in sleep, as well as exercise minutes, steps, and physiologic signals such as heart rate (HR) and respiratory rate (RR) [20]. Overall, these studies demonstrated that high-resolution data throughout pregnancy offer valuable insights into temporal and individual variability, as well as differences across different biobehavioral data, such as, sleep, temperature trends, cardiorespiratory, and activity.

This work aims to extend the study by Keeler et al [18] by conducting a large-scale mapping of biobehavioral changes across pregnancy—from preconception to postpartum—for pregnancies ending in loss (before 20 weeks), preterm birth, and term birth, using data from a large sample of 10,318 Oura Ring users. This retrospective observational study—the largest compilation of pregnancies using wearable data to date—aims to delineate the physiological and behavioral characteristics of pregnancy and to evaluate whether factors such as advanced maternal age and overweight status influence biobehavioral data trajectories during pregnancy. For example, existing literature indicates that advanced maternal age [21] and overweight/obesity [22] significantly increase the risk of adverse maternal outcomes; however, this study uniquely leverages wearable technology to provide continuous, real-world biobehavioral data across pregnancy, offering novel insights into how age and BMI impact sleep, activity, and cardiorespiratory patterns throughout gestation.

## Methods

### Dataset

A cohort of Oura Ring users was included in the study. Users were eligible if they self-identified as female (ie, sex assigned at birth), were pregnant between May 2023 and November 2024, and were aged 18 to 51 years. The date range was selected to ensure that participant data were collected using the latest Oura algorithms and Gen3 hardware. The final sample included 10,318 unique pregnancies. Among these, 5363 pregnancies led to live births (324 preterm and 5039 at term), while 4955 ended in loss before 20 weeks of gestation (eg, miscarriage or other early pregnancy losses).

### Ethical Considerations

This study involved secondary analysis of data from adult users of the Oura Ring, collected by Oura Health Oy (Oulu, Finland) in accordance with its Terms of Use [23] and Privacy Policy [24], which users consent to prior to data collection. By agreeing to the Privacy Policy, users consented to the use of their data for research and analytical purposes. All data used in this study were fully deidentified and aggregated in accordance with Oura’s internal data governance protocols. The research protocol was reviewed and approved by Oura’s internal science and legal teams, which oversee regulatory and ethical compliance for research activities. The study was deemed exempt from formal ethics board review as it involved only secondary analysis of anonymized, nonidentifiable data. Additional safeguards included restricted data access solely for verification and analytical accuracy. No compensation was provided to participants.

### Procedure

This is a retrospective observational study on Oura users who opted to use the Oura App’s Pregnancy Insights feature (see below) during pregnancy. Sleep, temperature trends, cardiorespiratory, and activity data from Oura Ring were accessed for a 64-week window encompassing the period from 8 weeks prior to the estimated start of pregnancy (the first day of the last menstrual period [LMP]) to 56 weeks following that.

**Figure 1:**
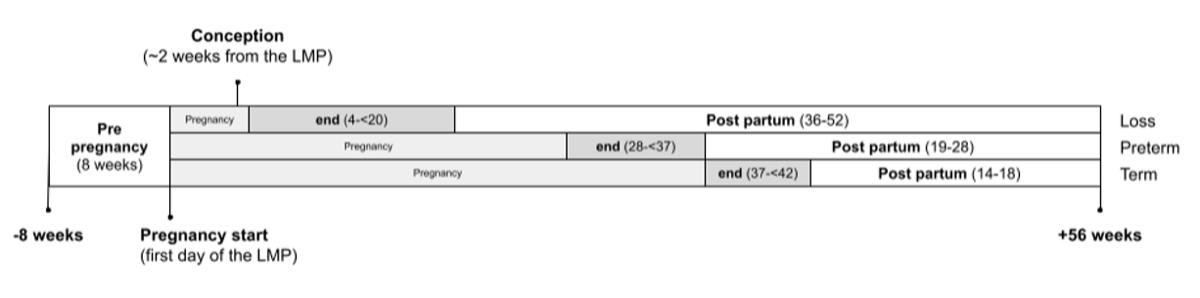
Schematics of the data collection timeline across pregnancies resulting in loss, preterm birth, and term birth are provided. The start and end of pregnancy, as well as the estimated time of conception, are highlighted in the graph. LMP: last menstrual period.

Participants were grouped based on self-reported pregnancy outcomes and the gestational age at pregnancy end, that is, pregnancies ending in loss versus live births (with live births further categorized into preterm and term births (Figure 1).

Gestational age was also used to classify pregnancy into 3 trimesters. Trimester 1 included weeks 0 days to 13 weeks 6 days of gestation. Trimester 2 ranged from 14 weeks 0 days to 27 weeks 6 days. Trimester 3 covered 28 weeks 0 days until 40 weeks 6 days of gestation.

### Oura Bio-Behavioral Data Collection

All participants wore an Oura Ring Gen3 (Oura Health Oy, Oulu, Finland), a noninvasive, water-resistant, and commercially available multisensor device that continuously measures finger blood flow, peripheral oxygen saturation, skin temperature, and movement via photoplethysmography green, red, and infrared LEDs, a negative skin temperature coefficient thermistor (±0.1 °C resolution), and 3D accelerometry. Oura Ring connects to mobile platforms via Bluetooth Low Energy, and data are transmitted to secure cloud-based servers for processing and storage. Oura Ring battery lasts up to 7 days.

By using proprietary algorithms, Oura Ring measures several health-related indices. Please refer to de Zambotti et al [25] for an overview of consumer wearable sleep metrics and their operationalization. A list of Oura Ring metrics used in this study is provided in Table 1.

**Table 1:** Oura Ring Gen3 metrics.

Measure	Description
Sleep	Daily Time in bed (hours), asleep (hours), awake (hours), and time spent in “light” sleep (equivalent of PSG^a^ N1+N2 sleep; % time asleep), “deep” sleep (equivalent of PSG N3 sleep; % time asleep) and rapid-eye-movement (REM) sleep (% time asleep), for the Oura classified main nocturnal sleep (>3h sleep duration). For each participant, single daily measures of overnight sleep measures were obtained. These metrics are calculated between the Oura Ring’s automatically detected onset and offset of nocturnal sleep windows, approximating individuals’ bedtimes and wakeup times via proprietary algorithms that combine features from PPG^b^, temperature trends, and accelerometer sensors, trained on expert-scored PSG sleep records. The performance of Oura Ring for sleep measurement has been evaluated across multiple peer-reviewed studies across different demographics, providing overall good agreement with the gold standard PSG [26-31]. In the most recent Oura-sponsored performance evaluation of Oura Ring Gen3 [29], Oura Ring achieved a sensitivity of 73.0% for wake, 76.7% for “light” sleep, 64.0% for “deep” sleep, and 72.6% for REM sleep, in healthy individuals.
Temperature	Daily peak sleep skin temperature (℃). For each of the participants, a single daily measurement of peak sleep skin temperature during sleep was obtained. Oura internal validation (unpublished company data) [32] shows good concordance of the skin temperature sensor with reference standards in both laboratory and free-living conditions.
Cardiorespiratory	Daily average sleep PPG-based HR^c^ (bpm) HRV^d^ (ms). For each of the participants and night, HR and HRV have been averaged across the Oura Ring’s classified main nocturnal sleep to provide a daily measure. Oura Ring Gen3 estimates HR at 30s resolution and time-domain HRV, that is, the root mean square of successive differences between normal heartbeats (RMSSD) for consecutive 5-minute windows during the nocturnal sleep. RMSSD captures the high-frequency component of HRV reflecting vagal autonomic nervous system function. Please see Shaffer and Ginsberg [33] for an overview of HRV assessment and meaning. As a note, the term HRV is used for consistency, considering that Oura Ring measures pulse rate variability from PPG signal, which reflects variation in blood flow and does not directly measure heart’s electrical depolarization signal. Average-per-night test comparing Oura Ring versus reference standard electrocardiogram-based HR and HRV metrics, shows good level of concordance with low mean biases [34]. Daily average respiratory rate (RR; breaths/min). RR is measured by computing PPG-HR time series data during nocturnal sleep. For each of the participants and night, RRs have been averaged across Oura classified main nocturnal sleep to provide a daily measure.
Activity	Daily total steps (number). Oura Ring calculates the number of daily steps within the time period spanning across the 24h. For each of the participants, a single daily measure of total steps was obtained. Oura activity data show good concordance for laboratory and free-living testing when compared with reference standards, particularly when considering group-level comparisons [35].

^a^PSG: polysomnography.

^b^PPG: photoplethysmography.

^c^HR: heart rate.

^d^HRV: heart rate variability.

### Oura App and the Pregnancy Insights Feature

The Oura App Pregnancy Insights feature is designed to provide data-driven information (how sleep and other biometrics change with pregnancy) and in-app education to help users navigate their pregnancy. The feature tracks gestational age, providing weekly updates about physiological changes detected by the Oura Ring. Pregnancy Insights requires a user to turn on the feature in the “Women’s Health” settings of the app, confirming pregnancy. The feature enables self-report of estimated due date and first day of the last period, as well as date of birth or pregnancy loss.

Age, prepregnancy height and weight, and country of residence have been extracted from the Oura App users’ profiles. BMI has been calculated as weight (kg) × height (m)^–2^.

### Data Preprocessing

#### Oura Ring Data

This step included the generation of “local” date-time indexing, removal of duplicate time points, and for sleep, temperature trends, and cardiorespiratory data, time points not belonging to main sleep periods (eg, naps, diurnal sleep) have been removed. In addition, pregnancies with less than 40% of Oura Ring data available [18], for prepregnancy and for each trimester during pregnancy, were excluded.

#### Identifying Pregnancy Start and End Date for Pregnancies Leading to Birth and Early Pregnancy End

Pregnancy start (standardized point for estimating gestational age) is operationalized as the first day of the LMP, which differs from conception (biological event), that is, the moment when the sperm fertilizes the egg, typically occurring around 14 days after the start of the LMP in a standard 28-day cycle. Pregnancy start is computed based on self-reported expected due date if known (expected due date minus 280 days; 5090/10,318, 49.3% pregnancies) or directly based on their self-reported first day of their last period (5228/10,318, 50.7% pregnancies). Pregnancy end was operationalized based on self-reported data, either as a birth date or as an early pregnancy loss.

### Statistical Analysis

A first statistical analysis was conducted to evaluate patterns of change in Oura bio-behavioral data throughout pregnancy, specifically focusing on pregnancies leading to term births. All bio-behavioral data were first aligned to the estimated start of pregnancy. The analysis was performed on average weekly bio-behavioral data from Oura, encompassing both the pregnancy and postpartum periods. To account for inherent interindividual variability in baseline physiological levels, each metric was normalized to a prepregnancy baseline. This baseline was defined as the average value over the 4 weeks preceding the estimated pregnancy start. Normalization was performed by calculating Z-scores, representing the number of SDs a value deviated from the mean of its respective prepregnancy baseline. This standardization allowed for a more focused capture and comparison of trends and patterns of physiological change throughout pregnancy across the cohort.

To appropriately account for the longitudinal within-subject correlation structure inherent in repeated measurements from the same individual, a generalized estimating equation (GEE) statistical modeling framework was used [36]. An exchangeable working correlation structure was specified for the GEE. This choice assumes a constant correlation between any 2 measurements within the same individual, regardless of the time interval between them, and is commonly used for its robustness. The GEE framework provides consistent estimates of population-averaged effects and computes robust standard errors, which are valid even if the assumed working correlation structure is not perfectly specified.

The nonlinear relationship between each normalized physiological metric and gestational age (our time variable) was modeled using B-spline basis functions. The specific flexibility of the curve was determined by using 8 degrees of freedom for the B-spline. This choice of degrees of freedom was guided by a balance between capturing complex nonlinear patterns (assessed through visual inspection of predicted fits) and maintaining statistical stability.

Subsequently, the impact of continuous covariates, specifically maternal age and prepregnancy BMI, as well as their interactions with gestational age, was investigated. This was achieved by extending the primary GEE model to include corresponding B-spline terms for these covariates and tensor product spline interaction terms with gestational age. These tensor product splines allowed for the characterization of how the nonlinear trend of each physiological metric over gestational age might vary across different levels of age and BMI.

The statistical significance of effects was assessed using Wald tests. First, for the gestational age-only model, a joint Wald test was performed on the set of B-spline coefficients for gestational age. A significant *P* value indicated a statistically significant nonlinear population-level trend in the physiological metric throughout pregnancy. Then, for models incorporating maternal age and BMI, joint Wald tests were conducted on the sets of coefficients corresponding to their main effect B-spline terms and interaction terms with gestational age. A statistically significant *P* value for these interaction terms indicated that maternal age or BMI had a significant moderating effect on the physiological trend over gestational age.

All tested effects (including the main trends over gestational age, and the main and interaction effects of age and BMI) were found to be statistically significant (*P* values <.05). However, it was observed that the magnitude of some of these effects (ie, the effect size) was small in practical terms, which was further explored through visualization of predicted trends and quantification of absolute differences over each trimester.

A second statistical analysis was conducted to evaluate differential trajectories in Oura bio-behavioral data between pregnancies ending before 20 weeks and those resulting in term live births, focusing on the period immediately preceding early pregnancy end. Specifically, we investigated how pregnancy outcome influenced physiological metrics during the 28 days leading up to the day of early pregnancy end. Prior to analysis, data underwent preprocessing, including linear interpolation for missing values and subsequent normalization via z-scoring with respect to each individual’s prepregnancy baseline (defined as the average over the 4 weeks preceding pregnancy start). For each pregnancy that ended before 20 weeks, we extracted bio-behavioral data for the 28 days immediately preceding the end of pregnancy. To establish a time-matched control, data from a randomly sampled pregnancy resulting in a term live birth were selected for the equivalent gestational age (eg, if a pregnancy end occurred at 7 weeks 1 day estimated gestational age, data from 3 weeks 2 days to 7 weeks 1 day were extracted for both the early pregnancy end case and its matched control).

A GEE model, like that used in the first analysis, was applied. In this model, the day relative to pregnancy end served as the primary independent variable to predict each normalized physiological metric, while simultaneously controlling for pregnancy outcome (pregnancy end before 20 weeks or term live birth). The nonlinear relationship between each normalized physiological metric and the day relative to a pregnancy ending before 20 weeks was modeled using a B-spline with 3 degrees of freedom. The model also included a linear term for pregnancy outcome and a linear interaction term between pregnancy outcome and the day relative to early pregnancy end. To determine if the physiological trajectory for pregnancies resulting in loss was statistically different from the trajectory for pregnancies resulting in a term live birth, we assessed the overall effect of pregnancy outcome using a joint Wald test on both the linear term for pregnancy outcome and the linear interaction term. Effect sizes were quantified as the mean difference between the modeled responses for pregnancies resulting in term live birth and those ending before 20 weeks, calculated across 2 distinct periods: the entire 28 days preceding early pregnancy end, and specifically during the 7 days immediately prior to the early pregnancy’s end.

All models were implemented using the Python package statsmodels [37]. This study is reported in accordance with the Strengthening the Reporting of Observational Studies in Epidemiology (STROBE) guidelines. A completed STROBE checklist is provided in Multimedia Appendix 1.

## Results

### Demographics and Pregnancy Characteristics

Participants in the study had a mean age of 32.2 (SD 4.5) years and a mean BMI of 25.3 (SD 5.4) kg/m², based on data collected prior to pregnancy.

Participants in the study represented various regions, including the United States (7398/10,318, 71.7% participants), the European Union (1104/10,318, 10.7% participants), the United Kingdom (351/10,318, 3.4% participants), Canada (320/10,318, 3.1% participants), Australia (155/10,318, 1.5% participants), and other countries (371/10,318, 3.6% participants). For 619 out of 10,318 (6.0%) participants, the location was unknown.

Distribution of Oura Ring sizes in the study population were the following: Size 6 (856/10,318, 8.3% participants), Size 7 (2270/10,318, 22% participants), Size 8 (3281/10,318, 31.8% participants), Size 9 (2384/10,318, 23.1% participants), Size 10 (1011/10,318, 9.8% participants), Size 11 (382/10,318, 3.7% participants), Size 12 (103/10,318, 1% participants), and Size 13 (31/10,318, 0.3% participants).

The distribution of pregnancies as a function of the estimated gestational age at which the pregnancy ended, for pregnancies ending in loss, preterm birth, and term birth, is provided in Multimedia Appendix 2. Self-reports of early pregnancy end did not differentiate between types of loss, such as spontaneous abortion, ectopic pregnancy, molar pregnancy, or pregnancy termination; therefore, this study does not adjust for the specific etiology of pregnancy loss. The distribution of participants’ age and BMI for pregnancies ending in loss, preterm birth, and term birth is provided in Multimedia Appendix 3.

Prepregnancy age (Kruskal-Wallis, *P*=.44) and BMI (Kruskal-Wallis, *P*=.08) did not significantly differ between pregnancies ending in loss, preterm, or term birth.

### Pattern of Changes in Oura Bio-Behavioral Data Throughout Pregnancy in Pregnancies Leading to Term Births

#### Overview

**Figure 2:**
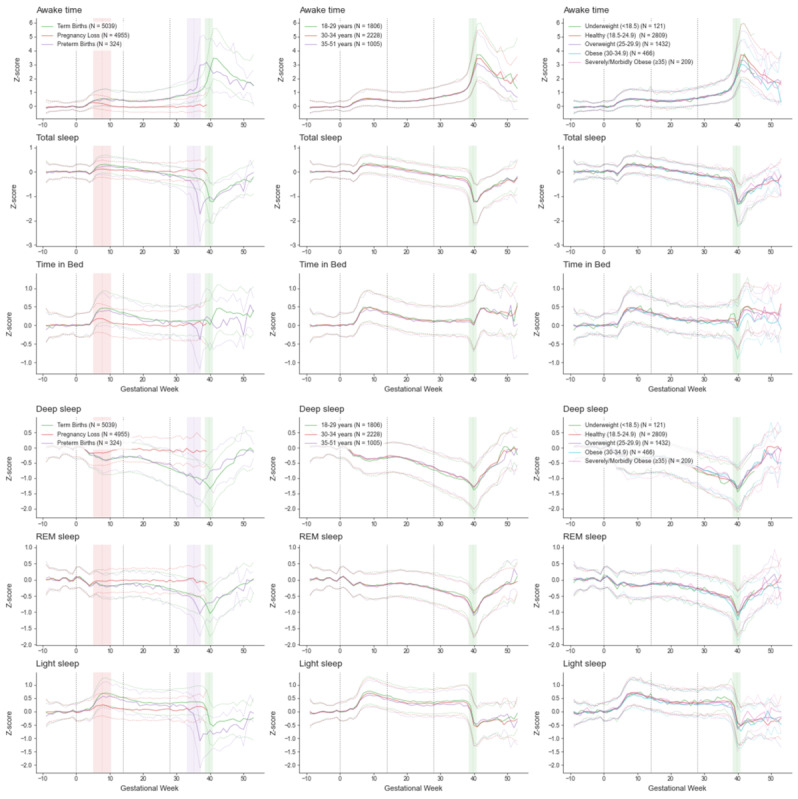
Changes in Oura sleep data throughout pregnancy. Data (Z-scores - normalized data based on prepregnancy individuals’ baseline) are plotted as the weekly median (thick line) and the 50th percentile range, separately for pregnancies leading to loss (N=4955), preterm births (N=324), and term (N=5039) births. Gestational age 0 (the start of pregnancy) is defined as the first day of the last menstrual period. The red, purple, and green vertical bars delineate the mean pregnancy end and one SD for pregnancy loss, preterm birth, and term birth, respectively.

**Figure 3:**
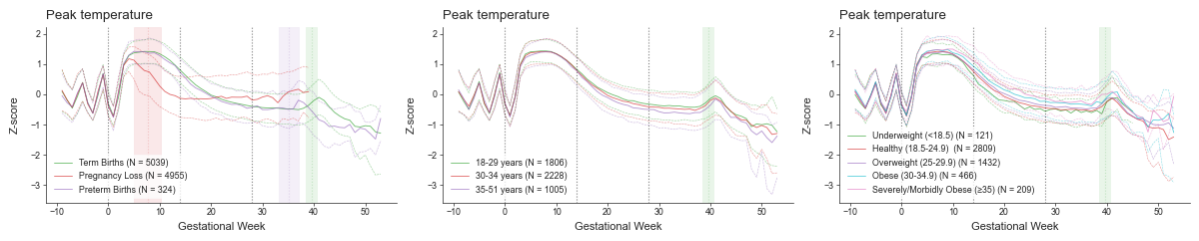
Changes in Oura temperature trends data throughout pregnancy. Data (Z-scores - normalized data based on prepregnancy individuals’ baseline) are plotted as the weekly median (thick line) and the 50th percentile range, separately for pregnancies ending in loss (N=4955), preterm births (N=324), and term (N=5039) births. Gestational age 0 (the start of pregnancy) is defined as the first day of the last menstrual period. The red, purple, and green vertical bars delineate the mean pregnancy end and one SD for pregnancy loss, preterm birth, and term birth, respectively.

**Figure 4:**
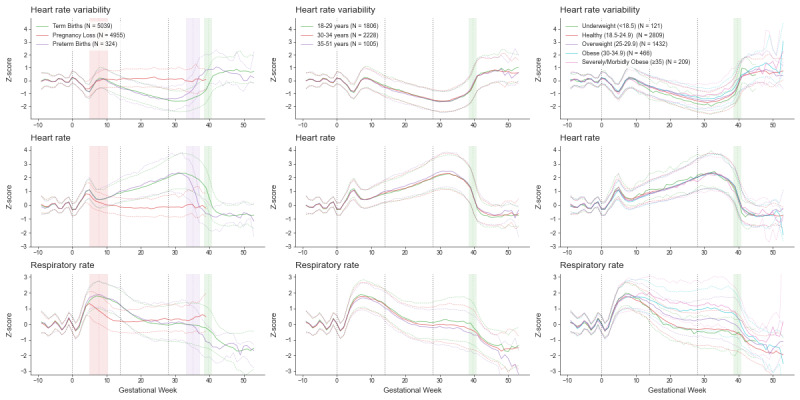
Changes in Oura cardiorespiratory data throughout pregnancy. Data (Z-scores - normalized data based on pre-pregnancy individuals’ baseline) are plotted as the weekly median (thick line) and the 50th percentile range, separately for pregnancies ending in loss (N=4955), preterm births (N=324), and term (N=5039) births. Gestational age 0 (the start of pregnancy) is defined as the first day of the last menstrual period. The red, purple, and green vertical bars delineate the mean pregnancy end and one SD for pregnancy loss, preterm birth, and term birth, respectively.

**Figure 5:**
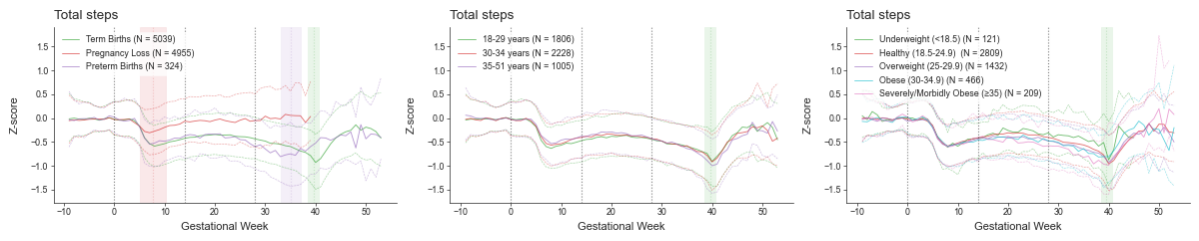
Changes in Oura activity data throughout pregnancy. Data (Z-scores - normalized data based on prepregnancy individuals’ baseline) are plotted as the weekly median (thick line) and the 50th percentile range, separately for pregnancies ending in loss (N=4955), preterm births (N=324), and term (N=5039) births. Gestational age 0 (the start of pregnancy) is defined as the first day of the last menstrual period. The red, purple, and green vertical bars delineate the mean pregnancy end and one SD for pregnancy loss, preterm birth, and term birth, respectively.

Absolute changes in Oura data throughout pregnancy are provided in Multimedia Appendix 4. The number of women contributing valid data at each gestational week is shown in Multimedia Appendix 5. Detailed results for the GEE model are provided in Multimedia Appendices 6 and 7. Changes in normalized Oura bio-behavioral data throughout pregnancy are displayed in Figure 2 (sleep), Figure 3 (temperature trends), Figure 4 (cardiorespiratory), and Figure 5 (activity).

While analyses focus on term births, the figures provide a visual comparison with pregnancies ending in loss and preterm birth. Pregnancies ending in loss (occurring between estimated gestational ages of 4 to 20 weeks) exhibit significant deviations from the trajectories of pregnancies resulting in birth at the same gestational age. In contrast, pregnancies resulting in preterm birth closely mirror Oura data trajectories of term births, except that the physiological changes associated with birth occur at an earlier gestational age.

#### Sleep

GEE results indicate a significant gestational age main effect for time in bed, time asleep, and time awake at night (all *P* values <.001). From the onset of pregnancy, time in bed, time asleep, and time awake all rise and reach a first peak around week 9. They then decrease and stabilize in the second trimester. In the third trimester, while time in bed returns to approximately pre-pregnancy level, time awake remains elevated and time asleep reduced. Around birth, sleep patterns change, with time in bed and time awake increasing to their peaks, while time asleep decreases to its lowest point, all occurring at week 40. Notably, participants’ time spent awake at around birth exceeds 3 SDs over baseline. GEE results indicate statistically significant gestational age-by-maternal-age and gestational age-by BMI interaction for each sleep metric (all *P* values <.01). However, the effect sizes are relatively modest except for differences following birth. We do see a slightly increased time asleep in younger compared with older participants from week 9 up to birth, with respect to prepregnancy level.

Similarly, significant gestational age main effects were observed for sleep stages (all *P* values <.001). Deep and rapid-eye-movement sleep both decreased throughout pregnancy, reaching a nadir around birth before recovering postpartum. Light sleep increased during the first trimester, reaching a peak around week 9, remained stable for most of pregnancy, and then declined postpartum. While statistically significant gestational age-by-maternal-age and gestational age-by BMI interactions were also found for these sleep stages (all *P* values <.01), their effect sizes were relatively modest. A subtle increase in light sleep and a decrease in deep sleep were noted in younger participants compared with older participants from week 9 until birth, relative to prepregnancy levels. There was a slight increase in rapid-eye-movement sleep from around week 33 until birth for younger participants compared with older participants.

#### Temperature Trends

GEE results indicate a significant gestational age main effect (*P* value <.001). From the start of pregnancy, temperature trends increase sharply at around conception (around 2-week gestational age), until it reaches a max peak around 9-week gestational age. It then gradually decreases to below prepregnancy levels. Around birth, temperature trends slightly increase, peaking at week 41, and afterward, it drops again below prepregnancy baseline levels. There are statistically significant but modest effects associated with age and BMI (all *P* values <.001). While the peak in temperature around week 9 is roughly the same across age groups, we do see a slightly less pronounced downward trend in temperature during the second and third trimesters for younger compared with older participants. The temperature peak around week 9 is slightly lower for participants with lower BMI compared with those with higher BMI, and there is a more significant drop in temperature during the second trimester for lower compared with higher BMI participants. This difference between lower and higher BMI participants persists through the third trimester.

#### Cardiorespiratory

GEE results indicate a significant gestational age main effect for HR and heart rate variability (HRV), as well as statistically significant interactions with age and BMI (all *P* values <.001). From the beginning of pregnancy, HR rises and reaches a first peak at week 5. After a small trough at week 9, it continues rising, peaking at week 32, then drops sharply at birth and stabilizes in the postpartum period. The HRV trajectory mirrors the opposite pattern of HR. Older participants exhibit a slightly larger rise in HR in the second and third trimesters (until approximately week 33), and have a slightly larger drop in HRV between the end of the first trimester and the first half of the second trimester (approximately from week 12 through week 24). Participants with lower BMI experienced a greater drop in HRV during the second trimester compared with those with higher BMI.

GEE results indicate significant gestational age main effect, gestational age by maternal age interaction, and gestational age by BMI interaction for RR (all *P* values <.001). From the onset of pregnancy, RR increases, peaking at 9 weeks of gestational age. It then steadily declines, stabilizing during the second and third trimesters. Following birth, RR experiences a sharp drop, falling below baseline levels. Interaction effects indicate that after reaching the first peak, RR decreases less in participants with a higher BMI and in younger participants.

#### Activity

GEE results indicate significant gestational age effects for total numbers of steps (*P* values <.001). From the start of pregnancy, the number of steps decreases, reaching a trough at gestational week 8 and then increases slightly until 21 weeks of gestational age (middle of the second trimester) before decreasing again until birth, reaching the min level of activity. GEE results indicate significant interactions between gestational age and both maternal age and BMI for the total number of steps (all *P* values <.001). Though statistically significant, the effect size for the maternal age interaction is modest. There is a greater decrease in steps starting in the second trimester for participants with higher BMI compared with those with lower BMI. There is also a slightly larger drop in steps for younger participants starting at the trough at gestational week 8, with the difference between younger and older participants disappearing by the end of the second trimester.

### Oura Biobehavioral Signatures Preceding Pregnancy Loss

**Figure 6:**
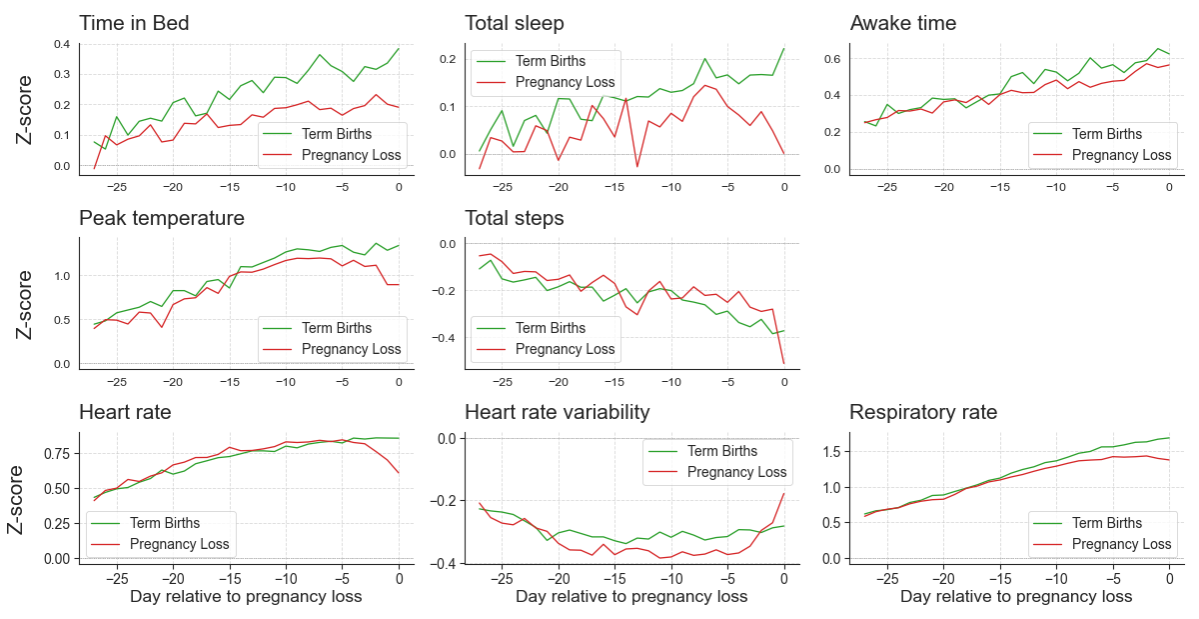
Changes in Oura sleep, temperature trends, activity, and cardiorespiratory data across 28 days preceding the end of pregnancy, for pregnancies ending in loss (N=4955), compared with Oura data trajectories matching the same gestational age, for a randomly chosen pregnancy leading to birth (N=4955).

Detailed results for GEE are provided in Multimedia Appendix 8. Changes in Oura bio-behavioral data for 28 days preceding and following the end of pregnancy are displayed in Figure 6. The number of women contributing valid data at each gestational week is shown in Multimedia Appendix 5.

The GEE model, comparing the loss versus nonloss curves, shows that the time in bed starts decreasing about 2 weeks prior to pregnancy loss (*P* values <.001), while total sleep time starts to decrease one week before (*P* values <.001). Awake time drops around the same period (*P*=.033). Temperature trends also show a significant effect (*P* values <.001), with peak temperature trends decreasing a week before pregnancy loss. Average HR drops a few days prior to pregnancy loss (*P*=.005), and RR drops one week before loss (*P*=.019). Steps and HRV effects were not statistically significant in the GEE models.

## Discussion

In this comprehensive, real-world, observational, retrospective study, we analyzed data from 10,318 pregnancies, providing the first large-scale examination of multimodal physiological and behavioral metrics recorded across the entire pregnancy continuum—from prepregnancy through conception and into the postpartum period. The novelty of the study lies in the high-density analysis of key biobehavioral aspects of pregnancy (sleep, temperature trends, cardiorespiratory, and activity data), mapping their trajectories over time and highlighting the complex, domain-specific temporal dynamics that characterize the multitude of biological and behavioral changes accompanying a woman’s pregnancy journey.

Changes that women experience throughout pregnancy are largely driven by hormonal shifts and can be viewed as adaptive responses designed to support a successful pregnancy and birth while protecting the mother [38,39]. Our results indicate that during the first trimester, participants experienced significant sleep alteration, peaking at week 9 and being characterized by increased time in bed (≈ +30 min), asleep (≈ +15 min), and awake (≈ +15 min) compared with prepregnancy levels. These changes align with rising progesterone levels, which have both thermogenic and soporific effects and can promote sleepiness and increase total sleep time, but also contribute to sleep disruption. While sleep duration returned to baseline (or lower) in the second trimester, wake time remained elevated, likely due to physiological adaptation to pregnancy as well as known factors such as increased frequency of nocturnal urination [40]. In the third trimester, sleep disturbances worsened, possibly driven by increased physical discomfort, fetal movements, or additional increases in nocturnal urination. After birth, sleep deteriorated dramatically, with wake time rising by ≈90 min and time spent asleep dropping ≈70 min below prepregnancy levels, possibly reflecting infant care demands, postpartum hormonal shifts, and disrupted circadian rhythms [41].

Temperature changes were more pronounced, sustained, and occurred earlier than sleep changes, becoming evident by 4 weeks estimated gestational age (≈+0.3 °C above baseline), mapping early hormonal shifts, particularly rising progesterone, which is known to increase basal skin temperature via thermogenic effects. These changes persisted throughout the first trimester. In the second trimester, temperature trends declined as maternal adaptation mechanisms, such as increased blood volume and improved thermoregulation (eg, response to increased metabolic heat produced by the fetus) [38], took effect, stabilizing below baseline in the third trimester. At birth, a slight skin temperature rise (≈+0.1 °C) may reflect labor-associated metabolic demands (eg, increased physical activity with uterine contractions) and hormonal fluctuations, followed by a drop postpartum as pregnancy-related thermogenic effects subside [38]. The postpartum return to monthly cyclical temperature oscillations (ie, the resumption of menstrual cycles) is not observed within the analyzed time window, which is limited to a few months after birth for live births. Given that menstruation gradually resumes, varying among individuals and influenced by factors such as lactation practices and hormonal fluctuations—none of which are captured in the current dataset—this process remains beyond the scope of our analysis.

Changes in cardiorespiratory data are consistent with the dynamic cardiovascular adaptations that occur throughout pregnancy to support the increasing metabolic demands of both the developing fetus and the mother [42]. Oura HR and HRV, that is, indices reflecting autonomic modulation, show profound changes across gestational age; they exhibit inverse patterns, characterized by a biphasic fluctuation in the first trimester—an initial HR peak exceeding 4 bpm above baseline and an HRV drop of >10 milliseconds at week 5, followed by a trough at week 9. These changes suggest early significant maternal physiologic preparatory changes to pregnancy, that is, happening before the end of the embryonic period, with HR rises following systemic vasodilation [43-45]. During the second trimester, HR increases linearly, peaking at >10 bpm above baseline, while HRV declines, reaching a minimum of >15 milliseconds below baseline, possibly reflecting decreases in parasympathetic cardiovascular control, as evaluated via standardized autonomic tests [46]. In the third trimester, these trends begin shifting earlier, with a marked drop in HR and a rise in HRV around birth, ultimately approaching prepregnancy levels.

The pulmonary system undergoes significant mechanical and functional changes during pregnancy. Notably, ventilation and tidal volume increase by approximately 20%-50% from prepregnancy levels [47]. Changes in RR are less well-defined in the literature; when reported, increases are typically modest, ranging from 1 to 2 breaths per minute. In our study, RR shows a slight increase during the first trimester, peaking around 8 weeks at approximately 1 breath per minute above baseline. This initial rise may reflect the stimulating effect of progesterone. Subsequently, the RR decreases and stabilizes by the mid-second trimester, returning to prepregnancy baseline levels, and remains unchanged until birth. After delivery, the RR may drop below baseline levels as the body readjusts to the nonpregnant state.

Our data indicate a decline in step count during the first trimester of pregnancy, with a notable dip around 8 weeks—a reduction of approximately 2000 steps compared with prepregnancy levels. This decline may be attributed to a combination of physiological, psychological, and social factors driven by hormonal fluctuations and early maternal adaptations. For example, elevated levels of human chorionic gonadotropin contribute to common pregnancy symptoms such as nausea, dizziness, and fatigue, which may lead to reduced physical activity [48]. Additionally, other factors such as worsening sleep quality and increased psychological stress may further contribute to decreased activity levels during this period. Cardiovascular adjustments can also impact energy levels. Following a slight increase in the second trimester, physical activity declines again, reaching its lowest point near birth, with step counts dropping by more than 2500 steps. This reduced activity in the third trimester may be explained by factors such as reduced mobility due to the mechanical strain, such as increased lordosis and laxity of joints, as well as increased effort on the cardiorespiratory system to sustain exercise under peak cardiometabolic demand as the maternal body supports rapid fetal growth, increased oxygen consumption, and metabolic adaptations to prepare for labor [42].

The use of passive, continuous longitudinal data collection in free-living conditions during pregnancy remains limited. However, our findings align with recent studies using consumer-grade devices. For example, 2 studies—one involving a limited sample of 17 (88%) US Hispanic women wearing Oura Ring devices [49] and another with a convenience sample of 20 Finnish women using Garmin Vivosmart bands [50]—revealed similar overall trajectories in sleep duration, activity levels, and HR increases through pregnancy. Focusing on photoplethysmography-based HR and a comprehensive set of time-domain and frequency-domain HRV metrics, Sarhaddi et al [51] analyzed data collected from 58 Finnish pregnant women, including those with high-risk pregnancies, using the Samsung Gear Sport smartwatch. Like our results, their findings reveal measure-specific changes in HR and HRV. Additional similar studies include pregnancy analyses based on Ava Fertility Tracker data in 23 women [52] and WHOOP 2.0 data in 18 healthy women [53]. These studies vary in sample characteristics, methodology, and purpose (eg, narrow focus on specific events during pregnancy), relying on small sample sizes and often lacking early pregnancy and prepregnancy baseline data, making direct outcome comparisons challenging. Additionally, many of these studies were conducted during the COVID-19 pandemic, which may have influenced pregnant women’s behaviors throughout pregnancy.

Notably, recent analyses of wearable biobehavioral data in 2540 Fitbit users [19], primarily focused on sleep, and an analysis of 733 Apple Watch users [20] revealed similar temporal patterns across the health metrics examined. Likewise, Keeler et al [18] analyzed Oura-derived biobehavioral trajectories in 97 full-term pregnancies and 23 early pregnancy losses. Our analysis builds upon and extends these findings by offering significantly greater temporal resolution, analyzing a substantially larger and more diverse sample, and uniquely including pregnancies ending in loss or preterm birth. These contributions allow for a more comprehensive characterization of physiological changes across pregnancy and highlight early, modality-specific deviations associated with nonviable outcomes. Importantly, similar to others [18,20], our results did not show large variation in Oura biobehavioral trajectories throughout pregnancy as a function of factors such as maternal age and prepregnancy BMI status. This aspect needs to be further explored. Notably, our evaluation of BMI was limited to prepregnancy status, and it is likely that different weight trajectories during pregnancy—such as the rate and pattern of weight gain—may affect pregnancy biometrics.

In our observations, we detected changes in sleep and downward shifts in temperature, RR, and HR preceding the reported early end of pregnancy, with most signal deflections beginning approximately 7 days before the event. These biobehavioral changes suggest the potential utility of continuous monitoring for identifying early indicators of pregnancy failure. Specifically, we were unable to determine the underlying cause or classification of these outcomes (eg, spontaneous miscarriage, ectopic pregnancy, molar pregnancy) due to the lack of diagnostic or medical metadata. As such, while we observed biobehavioral deviations in pregnancies not resulting in live birth, these findings should be interpreted as preliminary and hypothesis-generating. Future studies incorporating clinical validation and diagnostic classification are critical to elucidate whether specific biobehavioral patterns reliably differentiate among types of pregnancy loss, and to explore their potential for early detection of adverse outcomes.

Several limitations should be acknowledged. A major limitation of this work is the lack of detailed information on sample characteristics, pregnancy complications, and other biopsychosocial factors, which limits our ability to fully understand the complex influences on trajectories of sleep, temperature trends, cardiorespiratory function, and activity patterns throughout pregnancy. In addition, the observational design of the study does not allow for control of external variables or inference of causal relationships between biobehavioral changes and maternal outcomes. This is a retrospective analysis of data from Oura Ring users who engaged with an in-app feature specifically developed to support women during pregnancy. The sample likely overrepresents individuals with access to wearable technology and higher levels of health literacy, introducing demographic bias and limiting generalizability—particularly to underserved or marginalized populations. As such, the sample may not reflect the characteristics of the general population. For instance, while 71.7% of participants are based in the United States, only 13% meet the criteria for obesity—substantially lower than the 40.3% prevalence reported by the US Centers for Disease Control and Prevention among adults aged 20 years and older [54]. Furthermore, several key variables were self-reported, including prepregnancy BMI and the date of pregnancy end (eg, birth or loss), increasing the potential for recall bias and misclassification. The absence of clinical validation limits the applicability of these findings in medical settings and underscores the need for complementary studies that integrate health records and diagnostic assessments to enhance the translational utility of wearable-based monitoring.

This study also has inherent technological limitations. Wearable devices may be subject to measurement error and limited clinical validation—including during pregnancy, which involves profound physiological changes. Device accuracy may fluctuate across the pregnancy continuum, potentially masking some underlying physiological effects. These and other limitations of consumer wearable technology have been extensively discussed in recent literature [25,55,56]. In this study, in an attempt to control for potential confounders, we limited variability in device model and algorithm version by including only Oura Ring Gen3 users following the latest algorithm updates.

Future studies would also benefit from a multimodal data collection approach that combines passive wearable monitoring with ecological momentary assessments in more diverse populations. This would enable contextualization through variables such as reproductive history, lifestyle habits, occupational and relationship status, perceived support, health conditions, light exposure, physical activity not captured by the wearable (eg, activities performed when the wearable is not worn or not detected by the device), medication use, weight changes, and the presence or treatment of sleep disorders (eg, sleep-disordered breathing, restless legs syndrome). At the same time, this will be essential to improve generalizability and ensure that findings are applicable to racially, socioeconomically, and geographically diverse populations. Such enriched datasets would allow for nuanced characterization of the individual pregnancy experience and support the development of predictive models for both adverse pregnancy outcomes and natural events such as labor onset or early indicators of abnormalities—factors known to influence maternal and infant morbidity and mortality.

While outside the scope of this study, emerging evidence also highlights the potential relevance of circadian biology in shaping pregnancy outcomes. The reproductive system is tightly coupled to circadian regulation, and disruptions in maternal circadian timing—such as those caused by irregular light-dark cycles or shift work—are associated with negative pregnancy outcomes [57,58]. The placenta exhibits circadian gene expression, and disturbances in maternal circadian alignment may interfere with fetal-maternal signaling, nutrient transfer, and developmental timing [59]. There is evidence of a relationship between disturbed sleep during and after pregnancy and negative pregnancy outcomes, such as depression [60], anxiety [61], and gestational diabetes [62]. Going forward, incorporating circadian-focused assessments using wearable devices may provide novel insight into modifiable risk factors affecting both maternal well-being and fetal development. Although this study did not examine circadian-related metrics, wearable-derived data streams—such as nighttime temperature rhythms, intraindividual variability in sleep timing, and rest-activity rhythm—may offer promising noninvasive proxies for circadian alignment [25]. Future work should explore whether these features can serve as digital biomarkers of circadian misalignment and help identify individuals at higher risk for adverse pregnancy outcomes.

To conclude, this comprehensive exploration provides valuable insights into the physiological trajectories of pregnancy within a real-world population—information that until now, has not been widely available. By integrating passive sensing data with clinical information on reproductive history, pregnancy progression, and outcomes, this approach has the potential to advance our understanding of maternal health, enabling more personalized monitoring and improved pregnancy care. Beyond the continuous, automatic collection of relevant health metrics, this method allows for the examination of patterns relative to an individual’s baseline, rather than solely determining whether a single data point falls within a population range, irrespective of personal variation.

## Data Availability

The dataset analyzed during this study is not publicly available due to privacy protections for participants, but may be made available, in deidentified form, at Oura’s sole discretion.

## Appendix

Multimedia Appendix 1 STROBE checklist - cohort studies.

Multimedia Appendix 2 Distribution of volume of pregnancies as a function of gestational age at which pregnancies end, for pregnancies ending in loss, preterm births and term births.

Multimedia Appendix 3 Distribution of age and body mass index (BMI) of participants with pregnancies ending in loss, preterm births and term births.

Multimedia Appendix 4 Absolute changes in Oura data (sleep, temperature trends, cardiorespiratory, and activity) throughout pregnancy. Data are plotted as the weekly median (thick line) and the 50th percentile range, separately for pregnancies ending in loss (N = 4955), preterm births (N = 324), and term (N = 5039) births. Gestational age 0 (the start of pregnancy) is defined as the first day of the last menstrual period. The red, purple and green vertical bars delineate the mean pregnancy end ± one standard deviation for pregnancy loss, preterm birth, and term birth, respectively.

Multimedia Appendix 5 The number of women contributing valid data at each gestational week for pregnancies ending in loss, preterm births and term births.

Multimedia Appendix 6 Detailed results for Generalized Estimating Equation (GEE) model for analyses evaluating pattern of changes in Oura bio-behavioral data throughout pregnancy, in pregnancies leading to term births. REM, Rapid-Eyes-Movement-Sleep. Maximum effect sizes are summarized per trimester; however, the statistical model was fit over the entire pregnancy duration.

Multimedia Appendix 7 Detailed results for Generalized Estimating Equation (GEE) model for analyses evaluating effect of Body Mass Index (BMI) and maternal age on Oura bio-behavioral data throughout pregnancy, in pregnancies leading to term births. Maximum effect sizes are summarized per trimester; however, the statistical model was fit over the entire pregnancy duration.

Multimedia Appendix 8 Detailed results for Generalized Estimating Equation (GEE) model for analyses evaluating pattern of changes in Oura bio-behavioral data for the 28 days preceding pregnancies ending in loss. The effect size is calculated over the 28 and 7 days preceding the pregnancy ending before 20 weeks and represents the average difference with respect to corresponding biometrics over the same period from randomly selected full-term pregnancies.

## Abbreviations

GEE: generalized estimating equation
HR: heart rate
HRV: heart rate variability
LMP: last menstrual period
RR: respiratory rate
STROBE: Strengthening the Reporting of Observational Studies in Epidemiology
