# Supplementary material for: Temporal Trajectories in Sleep, Temperature Trends, Cardiorespiratory, and Activity Metrics Measured via Oura Ring During Pregnancy: Large-Scale Observational Analysis
Source: JMIR Mhealth Uhealth. 2025 Oct 27;13:e80213. doi: 10.2196/80213 (PMC12603580; doi:10.2196/80213)
Supplement: Multimedia Appendix 1 [file mhealth_v13i1e80213_app1.docx]

**Table S1** Strengthening the Reporting of Observational Studies in Epidemiology (STROBE) checklist - cohort studies.

|  | **Item No** | **Recommendation** | **Page No.** |
| --- | --- | --- | --- |
| **Title and abstract** | 1 | (*a*) Indicate the study’s design with a commonly used term in the title or the abstract | ✓ 1-2 |
|  |  | (*b*) Provide in the abstract an informative and balanced summary of what was done and what was found | ✓ 2 |
| **Introduction** | | |  |
| Background/rationale | 2 | Explain the scientific background and rationale for the investigation being reported | ✓ 3-4 |
| Objectives | 3 | State specific objectives, including any prespecified hypotheses | ✓ 4 |
| **Methods** | | |  |
| Study design | 4 | Present key elements of study design early in the paper | ✓ 5-6 |
| Setting | 5 | Describe the setting, locations, and relevant dates, including periods of recruitment, exposure, follow-up, and data collection | ✓ 5-7 |
| Participants | 6 | (*a*) Give the eligibility criteria, and the sources and methods of selection of participants. Describe methods of follow-up | ✓ 5-6 |
|  |  | (*b*) For matched studies, give matching criteria and number of exposed and unexposed | N/A |
| Variables | 7 | Clearly define all outcomes, exposures, predictors, potential confounders, and effect modifiers. Give diagnostic criteria, if applicable | ✓ 6-9 |
| Data sources/ measurement | 8* | For each variable of interest, give sources of data and details of methods of assessment (measurement). Describe comparability of assessment methods if there is more than one group | ✓ 6-7 |
| Bias | 9 | Describe any efforts to address potential sources of bias | ✓ 7-9 |
| Study size | 10 | Explain how the study size was arrived at | ✓ 5 |
| Quantitative variables | 11 | Explain how quantitative variables were handled in the analyses. If applicable, describe which groupings were chosen and why | ✓ 7-9 |
| Statistical methods | 12 | (*a*) Describe all statistical methods, including those used to control for confounding | ✓ 7-9 |
|  |  | (*b*) Describe any methods used to examine subgroups and interactions | ✓ 7-9 |
|  |  | (*c*) Explain how missing data were addressed | ✓ 9 |
|  |  | (*d*) If applicable, explain how loss to follow-up was addressed | N/A |
|  |  | (*e*) Describe any sensitivity analyses | N/A |
| **Results** | | |  |
| Participants | 13* | (a) Report numbers of individuals at each stage of study—eg numbers potentially eligible, examined for eligibility, confirmed eligible, included in the study, completing follow-up, and analysed | N/A |
|  |  | (b) Give reasons for non-participation at each stage | N/A |
|  |  | (c) Consider use of a flow diagram | N/A |
| Descriptive data | 14* | (a) Give characteristics of study participants (eg demographic, clinical, social) and information on exposures and potential confounders | ✓ 9-10 |
|  |  | (b) Indicate number of participants with missing data for each variable of interest | ✓ 10, 27 |
|  |  | (c) Summarise follow-up time (eg, average and total amount) | N/A |
| Outcome data | 15* | Report numbers of outcome events or summary measures over time | N/A |
| Main results | 16 | (*a*) Give unadjusted estimates and, if applicable, confounder-adjusted estimates and their precision (eg, 95% confidence interval). Make clear which confounders were adjusted for and why they were included | ✓ |
|  |  | (*b*) Report category boundaries when continuous variables were categorized | ✓ 5-14 |
|  |  | (*c*) If relevant, consider translating estimates of relative risk into absolute risk for a meaningful time period | N/A |
| Other analyses | 17 | Report other analyses done—eg analyses of subgroups and interactions, and sensitivity analyses | ✓ 9-14 |
| **Discussion** | | |  |
| Key results | 18 | Summarise key results with reference to study objectives | ✓ 15-19 |
| Limitations | 19 | Discuss limitations of the study, taking into account sources of potential bias or imprecision. Discuss both direction and magnitude of any potential bias | ✓ 17-18 |
| Interpretation | 20 | Give a cautious overall interpretation of results considering objectives, limitations, multiplicity of analyses, results from similar studies, and other relevant evidence | ✓ 15-19 |
| Generalisability | 21 | Discuss the generalisability (external validity) of the study results | ✓ 15-19 |
| **Other information** | | |  |
| Funding | 22 | Give the source of funding and the role of the funders for the present study and, if applicable, for the original study on which the present article is based | ✓ 21 |
