# Supplementary figures and images for: Temporal Trajectories in Sleep, Temperature Trends, Cardiorespiratory, and Activity Metrics Measured via Oura Ring During Pregnancy: Large-Scale Observational Analysis
Source: JMIR Mhealth Uhealth. 2025 Oct 27;13:e80213. doi: 10.2196/80213 (PMC12603580; doi:10.2196/80213)

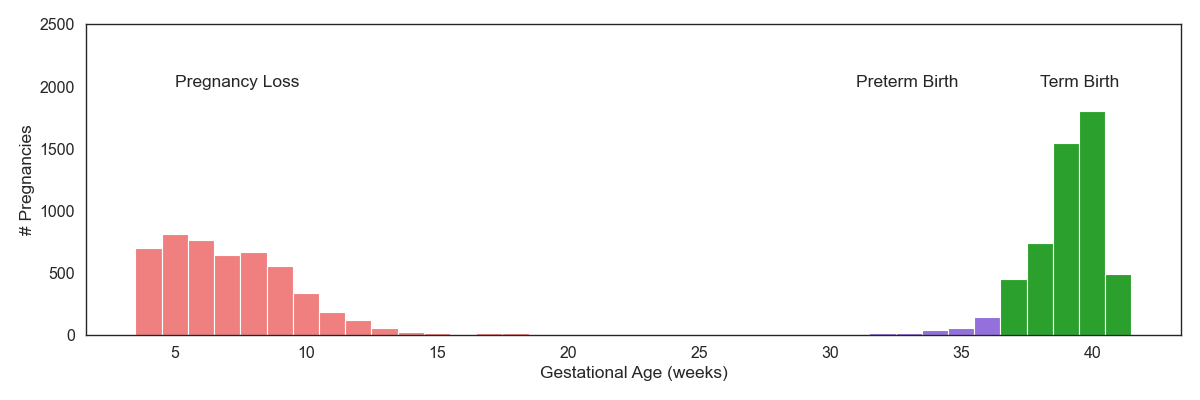

Supplement: Multimedia Appendix 2 [file mhealth_v13i1e80213_app2.png]

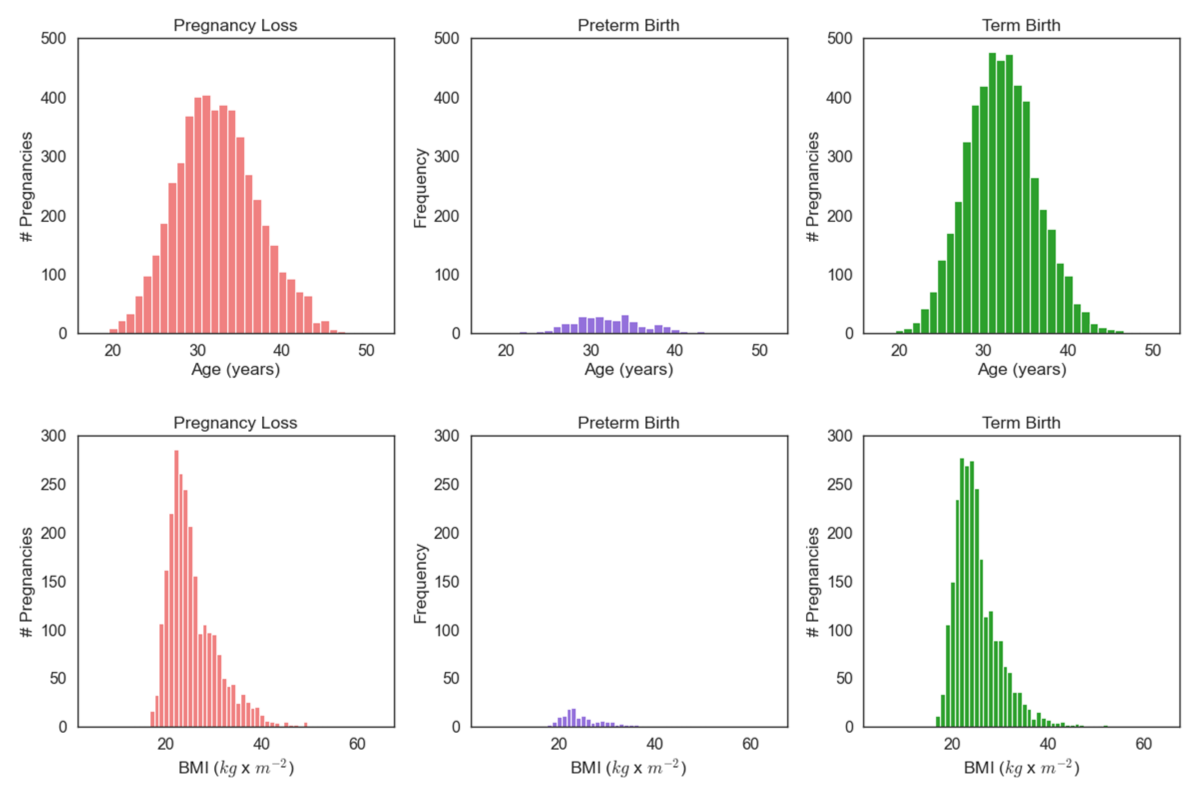

Supplement: Multimedia Appendix 3 [file mhealth_v13i1e80213_app3.png]

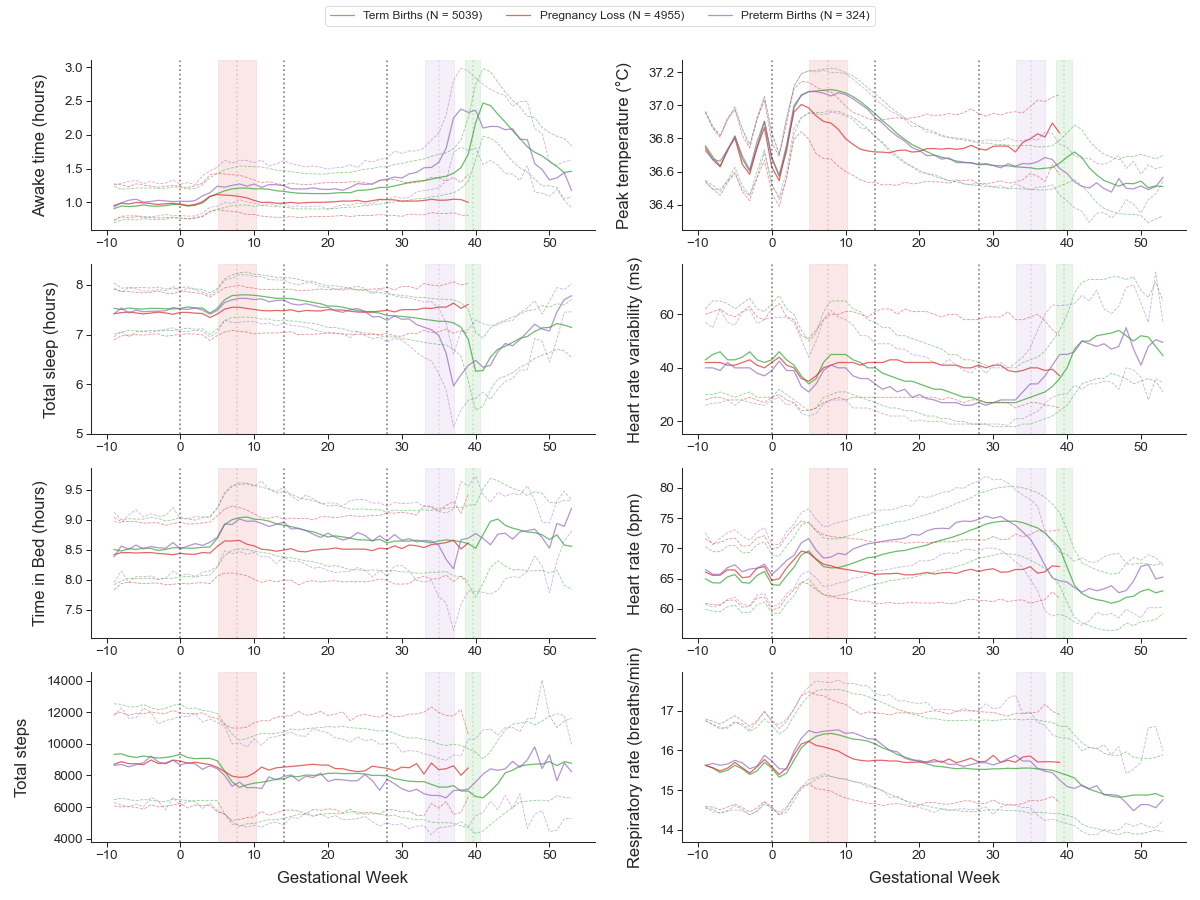

Supplement: Multimedia Appendix 4 [file mhealth_v13i1e80213_app4.png]

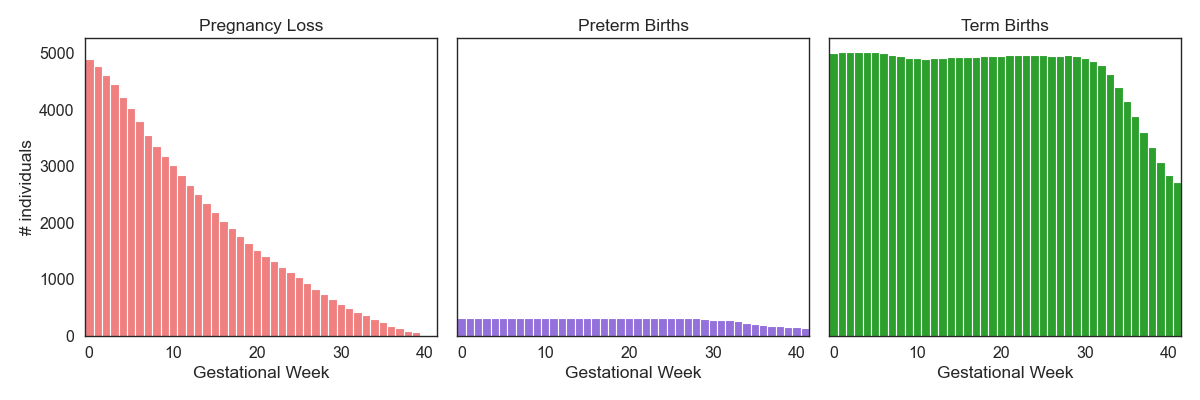

Supplement: Multimedia Appendix 5 [file mhealth_v13i1e80213_app5.png]
