# Supplementary material for: Temporal Trajectories in Sleep, Temperature Trends, Cardiorespiratory, and Activity Metrics Measured via Oura Ring During Pregnancy: Large-Scale Observational Analysis
Source: JMIR Mhealth Uhealth. 2025 Oct 27;13:e80213. doi: 10.2196/80213 (PMC12603580; doi:10.2196/80213)
Supplement: Multimedia Appendix 6 [file mhealth_v13i1e80213_app6.docx]

**Table S2** Detailed results for Generalized Estimating Equation (GEE) model for analyses evaluating pattern of changes in Oura bio-behavioral data throughout pregnancy, in pregnancies leading to term births. REM, Rapid-Eyes-Movement-Sleep. Maximum effect sizes are summarized per trimester; however, the statistical model was fit over the entire pregnancy duration.

| **Metric** (z-score) | ***P*-value** | **Maximum deviation from baseline in z-score** (by Trimester) | | | **Week of gestation with Maximum Delta** (by Trimester) | | |
| --- | --- | --- | --- | --- | --- | --- | --- |
|  |  | **T1** | **T2** | **T3** | **T1** | **T2** | **T3** |
| Time in bed | <.001 | 0.51 | 0.39 | 0.21 | 10 | 14 | 40 |
| Time asleep | <.001 | 0.3 | 0.26 | -0.85 | 11 | 14 | 40 |
| Time awake | <.001 | 0.77 | 0.74 | 2.74 | 9 | 26 | 40 |
| Time in light sleep | <.001 | 0.74 | 0.65 | 0.45 | 10 | 14 | 31 |
| Time in deep sleep | <.001 | -0.46 | -0.74 | -1.13 | 10 | 27 | 37 |
| Time in REM sleep | <.001 | -0.19 | -0.25 | -0.68 | 10 | 27 | 40 |
| Peak skin temperature | <.001 | 1.53 | 0.8 | -0.64 | 9 | 14 | 40 |
| Steps | <.001 | -0.52 | -0.43 | -0.61 | 10 | 14 | 36 |
| Heart rate | <.001 | 1.04 | 2.34 | 2.76 | 13 | 27 | 32 |
| Heart rate variability | <.001 | -0.3 | -1.54 | -1.7 | 13 | 27 | 30 |
| Respiratory rate | <.001 | 1.97 | 1.34 | -0.47 | 9 | 14 | 40 |
