# Supplementary material for: Temporal Trajectories in Sleep, Temperature Trends, Cardiorespiratory, and Activity Metrics Measured via Oura Ring During Pregnancy: Large-Scale Observational Analysis
Source: JMIR Mhealth Uhealth. 2025 Oct 27;13:e80213. doi: 10.2196/80213 (PMC12603580; doi:10.2196/80213)
Supplement: Multimedia Appendix 7 [file mhealth_v13i1e80213_app7.docx]

**Table S3** Detailed results for Generalized Estimating Equation (GEE) model for analyses evaluating effect of Body Mass Index (BMI) and maternal age on Oura bio-behavioral data throughout pregnancy, in pregnancies leading to term births. Maximum effect sizes are summarized per trimester; however, the statistical model was fit over the entire pregnancy duration.

| **Metric** (z-score) | **Effect Variable** | ***P*-value** | **Maximum Effect Size in z-score**  (by Trimester) | | | **Week of Gestation with Maximum Delta** (by Trimester) | | |
| --- | --- | --- | --- | --- | --- | --- | --- | --- |
|  |  |  | **T1** | **T2** | **T3** | **T1** | **T2** | **T3** |
| Time in bed | BMI | <.001 | 0.11 | 0.12 | 0.21 | 10 | 26 | 40 |
|  | age | 0.001 | 0.12 | 0.11 | 0.09 | 11 | 14 | 40 |
| Time asleep | BMI | <.001 | 0.1 | 0.12 | 0.2 | 13 | 24 | 40 |
|  | age | <.001 | 0.11 | 0.11 | 0.12 | 13 | 15 | 32 |
| Time awake | BMI | <.001 | 0.17 | 0.19 | 0.41 | 13 | 23 | 40 |
|  | age | <.001 | 0.08 | 0.1 | 0.24 | 0 | 22 | 40 |
| Time in light sleep | BMI | <.001 | 0.17 | 0.22 | 0.54 | 9 | 23 | 40 |
|  | age | <.001 | 0.2 | 0.21 | 0.22 | 10 | 24 | 39 |
| Time in deep sleep | BMI | <.001 | 0.16 | 0.2 | 0.24 | 12 | 27 | 32 |
|  | age | <.001 | 0.13 | 0.15 | 0.2 | 9 | 27 | 33 |
| Time in REM sleep | BMI | <.001 | 0.06 | 0.11 | 0.16 | 13 | 27 | 40 |
|  | age | 0.001 | 0.06 | 0.08 | 0.13 | 13 | 27 | 38 |
| Peak skin temperature | BMI | <.001 | 0.23 | 0.4 | 0.42 | 13 | 22 | 39 |
|  | age | <.001 | 0.12 | 0.28 | 0.28 | 2 | 26 | 28 |
| Steps | BMI | <.001 | 0.33 | 0.73 | 0.72 | 11 | 27 | 28 |
|  | age | <.001 | 0.2 | 0.2 | 0.28 | 13 | 14 | 39 |
| Heart rate | BMI | <.001 | 0.3 | 0.45 | 0.49 | 10 | 27 | 31 |
|  | age | <.001 | 0.12 | 0.24 | 0.16 | 13 | 19 | 28 |
| Heart rate variability | BMI | <.001 | 0.11 | 0.7 | 0.85 | 13 | 27 | 35 |
|  | age | <.001 | 0.2 | 0.25 | 0.13 | 13 | 16 | 40 |
| Respiratory rate | BMI | <.001 | 0.92 | 2.07 | 2.17 | 13 | 27 | 31 |
|  | age | <.001 | 0.28 | 0.44 | 0.71 | 13 | 27 | 34 |
