# Supplementary material for: Temporal Trajectories in Sleep, Temperature Trends, Cardiorespiratory, and Activity Metrics Measured via Oura Ring During Pregnancy: Large-Scale Observational Analysis
Source: JMIR Mhealth Uhealth. 2025 Oct 27;13:e80213. doi: 10.2196/80213 (PMC12603580; doi:10.2196/80213)
Supplement: Multimedia Appendix 8 [file mhealth_v13i1e80213_app8.docx]

**Table S4** Detailed results for Generalized Estimating Equation (GEE) model for analyses evaluating pattern of changes in Oura bio-behavioral data for the 28 days preceding pregnancies ending in loss. The effect size is calculated over the 28 and 7 days preceding the pregnancy ending before 20 weeks and represents the average difference with respect to corresponding biometrics over the same period from randomly selected full-term pregnancies.

| **Metric** (z-score) | **P-value** | **Average effect size**  (over 28 days preceding early pregnancy end) | **Average effect size**  (over 7 days preceding early pregnancy end) |
| --- | --- | --- | --- |
| Time in bed | <.001 | 0.09 | 0.14 |
| Total sleep time | <.001 | 0.06 | 0.09 |
| Awake time | 0.033 | 0.05 | 0.08 |
| Peak skin temperature | <.001 | 0.11 | 0.18 |
| Steps | 0.367 | -0.05 | -0.06 |
| Average heart rate | 0.005 | 0.00 | 0.05 |
| Average heart rate variability | 0.449 | 0.03 | 0.03 |
| Average respiratory rate | 0.019 | 0.08 | 0.16 |
